# High-Entry Vertebral Artery Variant during Anterior Cervical Discectomy and Fusion

**DOI:** 10.1155/2021/8105298

**Published:** 2021-07-24

**Authors:** Jay Moran, Joseph B. Kahan, Christopher A. Schneble, Michele H. Johnson, Shin Mei Chan, Jonathan N. Grauer, Daniel R. Rubio

**Affiliations:** ^1^Yale University School of Medicine, New Haven, CT, USA; ^2^Department of Orthopaedics and Rehabilitation, Yale University School of Medicine, USA; ^3^Department of Radiology and Biomedical Imaging, Department of Neurosurgery, Yale University School of Medicine, USA

## Abstract

Anterior surgical approaches to the cervical spine have allowed for treatment of common and complex pathologies with excellent outcomes. During the approach, complications can result from injury to the surrounding structures. The transverse processes usually protect the vertebral artery (VA) as it enters at C6 and courses cranially through the transverse foramina to C2 (referred to as the V2 segment). This is a case report of a patient who presented with myeloradiculopathy attributed to a C4-C5 disc herniation, severe canal stenosis, and marked bilateral neural foraminal stenosis. Preoperative imaging showed the right VA entering the C4 transverse foramen. This anatomic variant on a routine MRI led to further imaging and precautions when performing an uneventful anterior cervical discectomy and fusion (ACDF) at C4-C5. A high VA entry point into the transverse foramen above C6 could increase the risk of iatrogenic vascular injury in anterior approaches to the cervical spine. Rarely reported, the currently presented case describes a patient with a C4 right VA entry variant and highlights the importance of proper surgical planning.

## 1. Introduction

Anterior surgical approaches to the cervical spine are used to treat many cervical conditions, including spondylosis, disc herniations, and fractures [1]. A successful procedure should allow pathology to be addressed, leading to symptomatic relief and functional improvement while simultaneously minimizing complications [1, 2]. Injury to neighboring structures (aerodigestive tract, vasculature, nerves, etc.) can be devastating [3]. As such, a thorough understanding of the surrounding anatomy and its potential variations are necessary in order to minimize the risk of iatrogenic injuries [2, 4].

The current report will focus on the vertebral artery (VA), which plays a crucial role in contributing to the posterior cerebral circulation [4–6]. The vertebral arteries typically arise from the subclavian (V1 segment), ascend from C6 to C2 through the transverse foramina (V2 segment), and then exit the C1 transverse foramina and course lateral-to-medial along the superior aspect of the C1 posterior arch to form a single basilar artery (V3-V4 segments) [4, 6, 7]. The normal course of the VA is identical bilaterally.

The VA enters the C6 transverse foramen in the vast majority of individuals (90-95%) [6, 8, 9]. However, rare entry anomalies at C2, C3, C4, C5, and C7 have been described in the literature [6, 8–10]. In the case of a high-entry (above C6) VA, the artery courses relatively unprotected in the anterior neck: traversing cranially outside the bony protection of the transverse foramen [3, 6, 9]. Entry anomalies expose the “unprotected” VA and place it at potential risk for inadvertent injury with anterior cervical spine surgery [6, 8–10]. Thus, the preoperative identification of these VA anomalies can reduce the chance of iatrogenic injury [3, 11].

The purpose of the following case report is to describe a patient with a C4 VA entry abnormality who underwent a C4-C5 ACDF where identification of the anatomic variant helped avoid surgical complications. Secondarily, we provide a discussion on VA entry variations based on the literature.

## 2. Case Description

The patient provided consent for this case report. A 60-year-old female presented with acute-on-chronic neck and bilateral upper extremity pain without significant trauma or inciting event. Her arm pain was localized to the lateral arm and elbow bilaterally with associated paresthesias and numbness consistent with C5 radiculopathy. She described moderate (6/10) activity-related neck pain. She described progressive fine motor difficulty over the past months interfering with her occupation as a waitress.

On physical exam, she endorsed paresthesias affecting the left arm from the lateral arm to the lateral elbow on light touch. She had no evidence of objective weakness with strength testing of the upper or lower extremities. Gait evaluation revealed nonantalgic, unassisted ambulation, with significant difficulty performing heel-to-toe tandem gait. Additionally, she had positive upper motor neuron findings, including a positive Hoffman sign and Romberg test.

Prior to our evaluation, she had tried conservative measures with minimal relief. These included physical therapy and anti-inflammatory medications.

**Figure 1:**
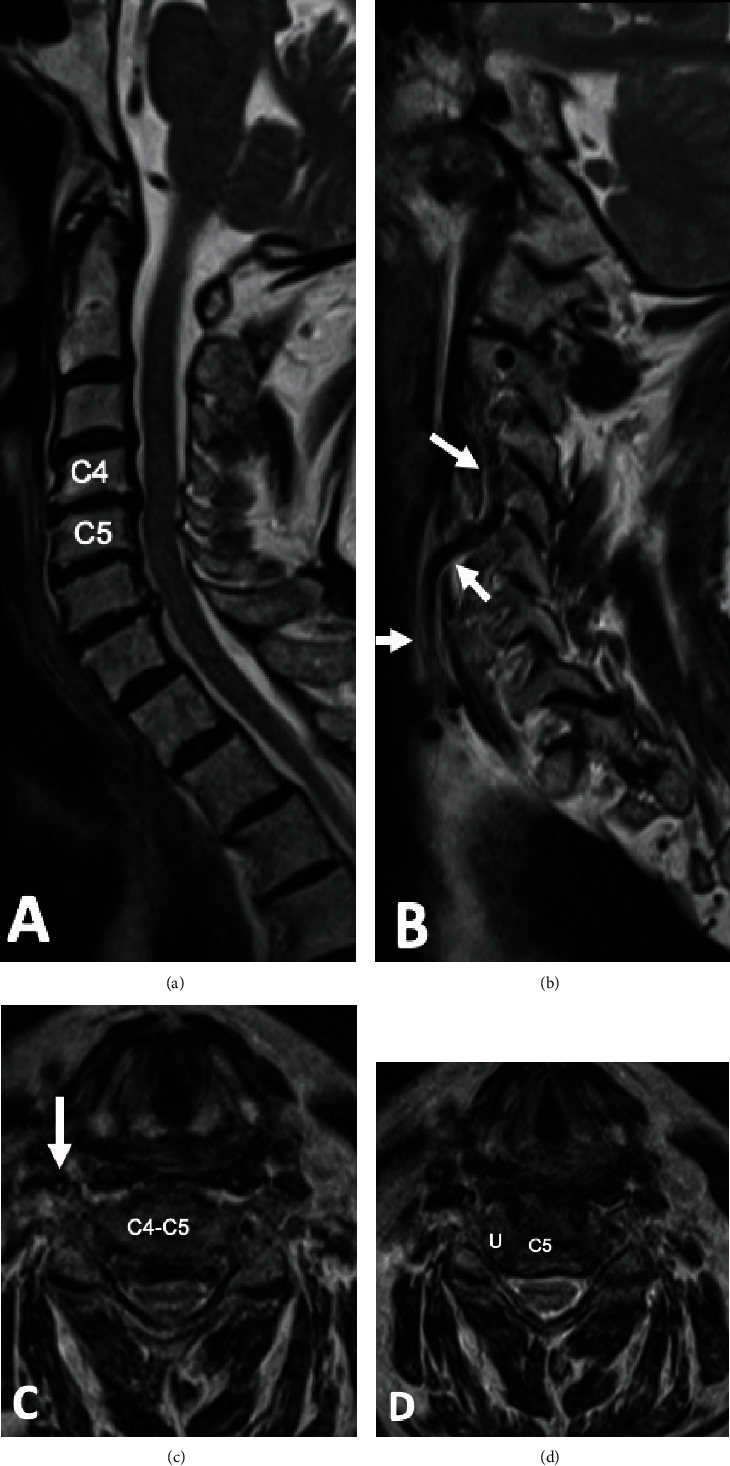
T2 images of the patient being presented. Midsagittal image demonstrates multilevel disc degeneration (a). Far right lateral sagittal image demonstrates anterior course of the right vertebral artery with entry into transverse foramen at C4 (white arrows) (b). Axial T2 images at the level of the C45 disc (c) and the uncovertebral joints (d) demonstrate central and bilateral neuroforaminal stenosis. Note the anterior position of the RVA (white arrow) at the C45 disc level (c). U: uncinate process.

A cervical MRI revealed multilevel degenerative changes throughout the cervical spine (Figure 1). Imaging showed disc osteophyte at C4-C5 with moderate to severe canal stenosis and marked bilateral neural foraminal stenosis.

**Figure 2:**
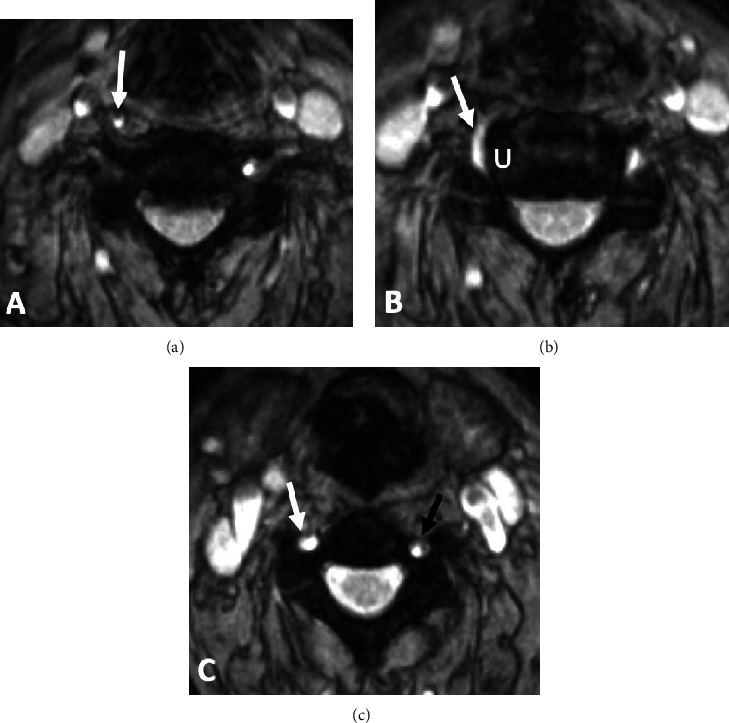
Axial MEDIC GRE of the patient being presented. The right-VA (RVA) courses anteriorly outside of the transverse foramen at C5 as compared to the left-VA (LVA) within the foramen (a). The RVA courses posteriorly to enter the transverse foramen at C4. Note the proximity of the RVA to the uncinate process (U) (b). At C4, both VA reside in the transverse foramina (RVA: white arrow; U: uncinate process; LVA: black arrow) (c).

**Figure 3:**
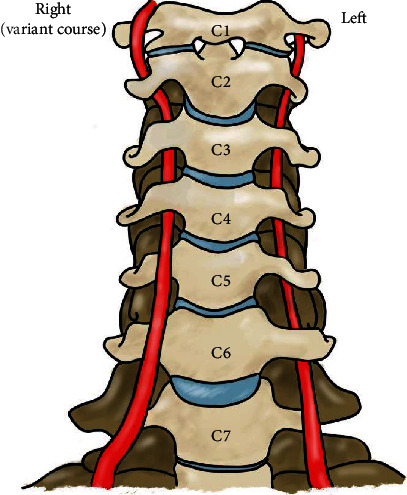
An anterior view of the cervical spine C7 to C1, with the RVA variant coursing outside of the transverse foramen, entering at C4. The normal course of the LVA is seen on the opposite side for reference.

Further review of MR imaging of the cervical spine revealed that the right VA exhibited anatomic variation in its course. This ascended cranially outside of the transverse foramen until entering at C4. Once in the C4 transverse foramen, the right VA resumed its typical course cranially, through the transverse foramina at C4 to C2 (Figure 2(c)). A pictorial description of the C4 VA high entry anomaly is depicted in Figure 3.

Overall, the patient's presentation suggested cervical C5 myeloradiculopathy (Nurick 2), as confirmed by advanced imaging with incidental right VA variation, entering the transverse foramen at C4-C5 disc level. Given this presentation and failure with conservative treatment, an anterior cervical decompression and fusion (ACDF) at C4-C5 was recommended. Additionally, the right vertebral artery variant anatomy was discussed with the patient, including the potential risk for arterial injury given the level of her expected discectomy and fusion.

A standard left-sided anterior cervical approach was performed, as described by Smith and Robinson [12]. The left-sided procedure is generally preferred in an anatomically normal patient, but it also allowed us to avoid anterior neck dissection around the VA variant course. If the VA variant was on the left side, a right sided approach would have been utilized instead.

After confirmation of the exposure of the C4-C5 disc space, the uncinate process was exposed bilaterally by elevating the longus colli muscle. On the left, the longus colli muscle was elevated via a combination of monopolar and bipolar cautery out to the lateral border of the uncinate process. On the right, given the VA anomaly, the longus colli muscle was elevated with careful blunt dissection using a Penfield 2 (NovoSurgical, Oakbrook, Illinois) to the lateral border of the uncinate process.

**Figure 4:**
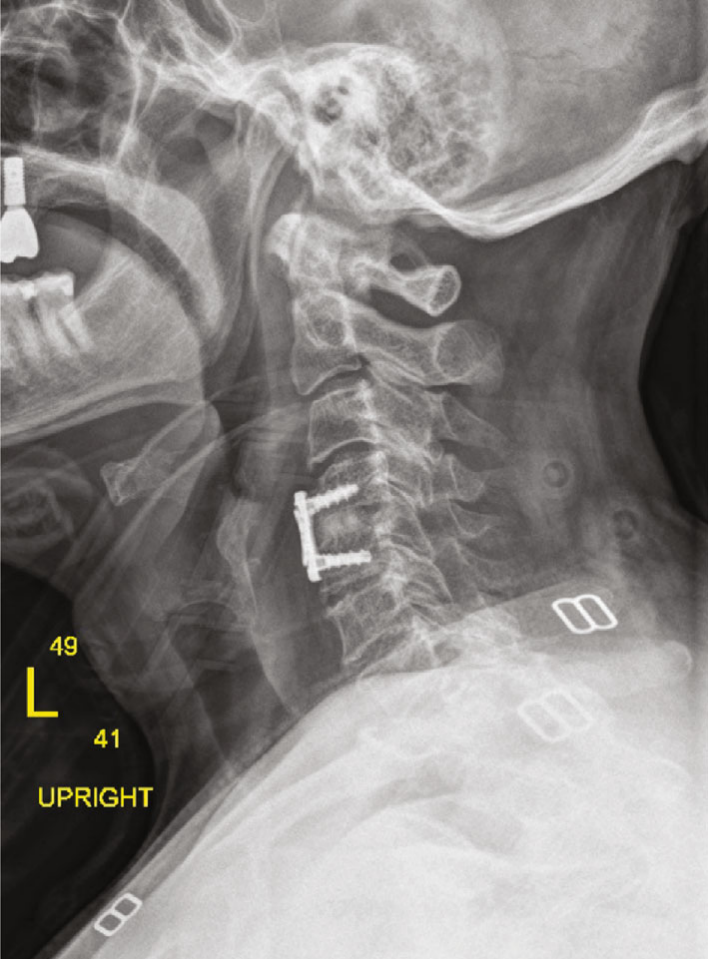
Lateral radiograph obtained six weeks postoperatively. Lateral radiograph reveals status-postanterior cervical discectomy with placement of allograft spacer and anterior cervical plate with variable angle screws.

The discectomy was performed at C4-C5 in standard fashion. After the decompression was complete, an anterior cervical locking plate (Medtronic Zevo, Memphis, TN) was placed (Figure 4). The patient had a successful postoperative course with no evidence of any vascular damage after surgery and at 1-year follow-up.

## 3. Discussion/Outcome

The VA is usually anatomically protected within the transverse foramen as it courses from C6 to C2 (V2 segment). As such, it is not often encountered in anterior cervical spine procedures. The purpose of this study is to report a case of a high right VA entry into the transverse foramen at C4 in a patient who underwent a C4-C5 ACDF that was completed without complication.

Prior anatomical studies have reported uncommon variations in the VA anatomy, including variations of the arterial origin, vascular loops, medial deviations into the vertebral bodies, and variable levels at which the VA enters the transverse foramen [6, 8–10]. Perioperative recognition of VA variant anatomy on cross-sectional imaging is important in order to avoid vertebral artery injury and potentially devastating surgical complications.

The most common level of entry for the VA into the transverse foramen is at C6, occurring in 90-95% of patients [6, 8–10]. Although rare, anatomical variations in the VA entry level have been reported in 6-10% of all cases [6, 8–10]. Bruneu et al. reported VA entries at the C3, C4, C5, or C7 level to represent 0.2%, 1.0%, 5.0%, and 0.8% of all cases, respectively [6].

Similarly, Matula et al. studied 402 VA entry points and reported that the VA entered the transverse foramen at C5 and C7 in 7% and 3% of all cases, respectively [10]. The majority of these VA entry anomalies occur unilaterally and show no preference to either the right or left side [6]. The right-VA C4 entrance anomaly described here has been reported to represent 0.5% of all cases [6, 8, 9].

Interestingly, Hong et al. reported that the area of the unfilled transverse foramen below the VA entrance was significantly smaller compared to the transverse foramina occupied by the VA on the contralateral side [9]. This suggests that VA entrance may play an important embryological role in the development of complete transverse foramens in the cervical spine [9].

VA injury in the setting of anterior cervical spinal surgery is a rare complication with an incidence of 0.3-0.5% [3, 13–15], though some authors have proposed that this number may be underestimated, as some VA injuries can go unrecognized and/or unreported in operative notes [3, 4, 6, 13–15]. Nevertheless, injury to the VA can lead to grave consequences including stroke, cardiac arrest, hemorrhage, neurological deficits, or death [3].

The incidence of nonvariant VA injury is thought to be relatively low during anterior cervical spine surgery because the V2 segment is protected by the transverse processes as it courses cephalad [9]. However, when the entry level is higher than C6, the VA courses anterior to the transverse process in the anterior neck in a relatively unprotected course outside the bony confines of the transverse foramen [6].

Guan et al. conducted a systematic review of 54 patients that experienced VA injuries in the setting of anterior cervical surgery [3]. Ten patients were reported to have VA anomalies, all of which were not identified preoperatively [3]. Similarly, Golfinos et al. conducted a retrospective analysis on 1,215 anterior cervical surgeries and reported that an anomalous VA course is a risk factor for perioperative arterial laceration [15]. When the VA lacks the typical bony protection as seen in entry point anomalies, the chances of iatrogenic VA injury increase, especially when inadvertent retraction or dissection of the longus colli muscle is performed [9]. It is imperative that the surgeon has a fundamental understanding of what to encounter when exposing the uncinate processes in anterior cervical spinal approaches [4, 6].

The best method to avoid unintentional VA injury is being aware of its anatomic course [3, 4, 6, 13–15]. Recognition of vertebral anomalies on conventional cross-sectional imaging, including MRI and CT with demonstration of the arterial position and/or size and shape of the transverse foramen, may be definitive as in this case. MRA and CTA may be adjunctive and most helpful in complex cases or in reoperation. Careful preoperative planning is necessary in cases of VA entrance anomalies in order to prevent life threatening sequelae [3, 4, 6].

During complex surgery, intraoperative CT and three-dimensional fluoroscopic navigation systems, as well as real time image guidance can aid in determining accurate anatomical variations [3]. Surgeons must be aware of these anomalies and accommodate accordingly with preoperative planning, careful dissection, and/or novel techniques [3].

## 4. Conclusion

In conclusion, the anterior approach is used to treat several pathologies involving the cervical spine. VA injuries can occur when anatomic variations exist; thus, it is important that VA position and course are studied on preoperative imaging. We presented a case of a rare high-entry VA variant in a patient with cervical myeloradiculopathy from a C4-C5 disc herniation and disc-osteophyte complex. Preoperative MRI revealed the right VA coursed anterior-to-posterior lateral to the C5 uncinate prior to entry in to the right C4 transverse foramen. Given the VA variant was identified preoperatively, careful exposure of the C4-C5 disc and dissection of the C5 uncinate resulted in successful C4-C5 ACDF without complication.
